# Exercise Training and Insulin Resistance: A Current Review

**DOI:** 10.4172/2165-7904.S5-003

**Published:** 2015-07-30

**Authors:** Tyler E Keshel, Robert H Coker

**Affiliations:** 1 Institute of Arctic Biology, University of Alaska-Fairbanks, Fairbanks, AK, USA; 2 Center for Translational Research in Aging and Longevity, Department of Geriatrics, University of Arkansas for Medical Sciences, Little Rock, AR, USA

**Keywords:** Weight loss, Inflammation, Metabolism

## Abstract

There is a general perception that increased physical activity will improve glucose homeostasis in all individuals. While this is an attractive concept, this conclusion may be overly simplistic and even misleading. The topic was reviewed extensively over 30 years ago and it was concluded that acute exercise enhances glucose uptake. However, in some cases the chronic influence of interventions utilizing exercise may have little effect on glucose metabolism. Moreover, insulin resistance often returns to near baseline levels within a couple of days following cessation of the exercise bout; leaving the overall effectiveness of the intervention in question. Since improving glucose homeostasis should be the focal endpoint of any intervention designed to mitigate the overwhelming degree of insulin resistance in individuals at risk for metabolic disease, it is essential to evaluate the key components of a successful approach.

## Introduction

Insulin resistance is commonly presented in older adults, but has become increasingly prevalent in all ages, including middle-aged individuals that are overweight and sedentary [1–8]. An excessive deposition of adipose tissue, defined as overweight (BMI 25–30 kg/m^2^) and obese (BMI ≥ 30 kg/m^2^), is a risk factor for the development of type 2 diabetes [7], and is associated with a reduced uptake of glucose [9]. However, the overall etiology of insulin resistance is quite complex. It is known that a disproportionate accumulation of subcutaneous and abdominal fat contributes to the desensitization of insulin receptors that is characterized by an inhibited uptake of glucose within skeletal muscle and an impaired ability to suppress endogenous glucose production [10–13]. From a simplistic standpoint, these physiological alterations may lead not only hyperglycemia, but also to exacerbations in fat deposition [10,11], promoting a chronic state of inflammation characterized by the release of pro-inflammatory cytokines [9,11,14]. As insulin resistance worsens, β-cells continually stimulate insulin production in an attempt to reduce overwhelming hyperglycemia, but remain ineffective and may influence an additional state of hyperinsulinemia [15].

To combat these deleterious consequences, the management of obesity has expanded. For example, the popularity of physical activity as a common intervention strategy may be due to its long-term effectiveness against insulin resistance when using the Oral Glucose Tolerance Test (OGTT) as a benchmark for improved glucose homeostasis [15]. The acute and chronic influence of physical activity has a multifaceted influence on glucose metabolism, including acute changes in contraction mediated glucose uptake and chronic changes in insulin-stimulated glucose uptake that may be mediated by a variety of mechanisms.

Since the translation of scientific investigation to clinical care and community engagement represents a tremendous responsibility (NCEP ATP III), recommendations for physical activity designed to mitigate the detrimental influence of insulin resistance must be precise in their application. From our perspective, the distinction between acute exercise, and chronic exercise training (with or without weight loss), or in conjunction with caloric restriction has not been clearly delineated. Therefore, the purpose of this review is to outline the key variables of physical activity that may have a different influence on the mitigation of insulin resistance, and how they might serve to ameliorate the clinical condition altogether.

## Search Method

The PubMed database was utilized to conduct a search in November of 2013-April 2015 that consisted of the following keywords: insulin resistance or insulin sensitivity each combined with exercise. Upon retrieval of over 5,000 publications, a year filtration was applied to identify studies between the years 2008 and 2013. An additional filter for “Free Full Text available” was applied, which yielded a total of 978 publications.

### Inclusion criteria/rationale

From a total of 978 identified publications, consideration for inclusion was dependent upon: 1) use of an exercise intervention and 2) whether insulin resistance was assessed before and after intervention (excluding fasting plasma glucose/insulin sampling). After reviewing each title, the total was reduced to 264 publications. Of the 260, the abstract of each was examined for: 1) subjects >30 years of age, 2) overweight to obese, according to body mass index (BMI >25 kg/m^2^), 3) subjects free of disease, including no previous diagnosis and no active treatments for cardiovascular disease, diabetes mellitus, and cancer, 4) subjects were not taking hormone replacement medications, or any medications that may have affected their metabolism, and 5) were nonsmokers. After the abstracts were reviewed, the 264 total was reduced to 158 publications that were considered for a full text review using the same criteria aforementioned. Review articles and references were then searched for additional primary studies up to the year 2014.

Since the primary aim of this review is the influence of exercise training on insulin resistance, the subject inclusion/exclusion criteria was adopted from majority of the investigations focused on this specific topic. Given the diversity of investigations with regards to the intervention and insulin resistance/sensitivity assessment tool, a narrative review was chosen to examine the components of the exercise and how they might influence changes in insulin sensitivity. Although multiple assessments for insulin resistance/sensitivity exist, comparisons will not be fully discussed in this review; as they are detailed elsewhere [16].

## Importance of Regional Body Composition

There seems to be a direct connection between obesity and insulin resistance, but is highly dependent upon regional fat deposition [9,12,15]. For example, in a study by Jenkins et al. 2011, mean baseline BMI between normoglycemic and prediabetic individuals was 27.9 ± 0.4 kg/m^2^ and 28.9 ± 0.6 kg/m^2^ respectively with no significant difference (p=0.18) between groups. However, the prediabetic group had a significantly (p=0.005) higher mean percentage of trunk fat (as determined by DXA scan) of 39.9 ± 1.2% vs. 35.7 ± 0.8% of the normoglycemic group. This translated into a mean baseline homeostasis model assessment for insulin resistance (HOMA-IR) score of 3.8 ± 0.2 for the prediabetic group versus a significantly more favorable HOMA-IR score of 2.8 ± 0.1 for the normoglycemic group. Therefore, the evaluation of regional fat distribution differentiation is important to evaluate since these areas of fat deposition may influence insulin resistance differently and exercise training may have a variable influence on specific fat depots [17].

### Exercise alone without weight loss

Exercise training without weight loss may enhance insulin-stimulated glucose disposal [17–21] but insulin resistance commonly returns to near baseline levels after cessation of the exercise [2,22–24], suggesting that activity without weight loss primarily affects variations in glycogen stores and glycogen synthase activity [25]. However, exercise training in the absence of weight loss may influence changes in body composition variables such as fat mass [2,19], visceral and subcutaneous adipose tissue [17,23,26], and body fat percentage [21,27], which may then translate into improvements in insulin-stimulated glucose disposal.

In an aerobic exercise intervention that utilized a progressive increase in intensity (i.e., 50 to 70% of VO_2max_) over a 6 month period with 3 exercise sessions per week, there was no weight loss but the intervention did promote significant reductions in intra-abdominal fat (−10 ± 4.0%) and subcutaneous fat (−15 ± 7.3%) which resulted in an approximate 5% reduction in glucose area under the curve (AUC) (Jenkins et al. 2011). Additionally, these changes in body composition promoted a 16% reduction in HOMA-IR scores within the same individuals (Jenkins et al. 2011). In a similar intervention that utilized vigorous intensity exercise (65% of VO_2peak_) but progressively increased the sessions per week (3 to 5 sessions) and duration per session (30 to 60 minutes), weight reduction did not occur. Nonetheless, the individuals in this particular study also demonstrated improvements in VO_2peak_ (+16 ± 4 ml/kg/min) that partially supported improvements in free fatty acid mobilization and oxidation [18].

It seems that there are similarities among the exercise interventions that observed insulin sensitivity changes in the absence of weight loss. For instance, in studies where investigators implemented aerobic exercise beginning with moderate intensity (50–60% of HR_max_ and 50% of VO_2peak_) but progressed successfully to a vigorous intensity (60–70% of HR_max_ and 70% of VO_2peak_) by the end of the intervention. Interestingly, they observed a significant reduction in waist circumference (111.8 ± 3.7 to 108.6 ± 4.2 cm^2^) and concomitant increases in mitochondrial density at the sarcolemmal (11.9 ± 1.6 to 15.9 ± 1.4%) and the intermyofibrillar (3.2 ± 0.4 to 5.3 ± 0.5%) levels. These changes occurred in conjunction with an additional improvement in plasma glucose at 120 minutes of an OGTT from 7.09 ± 0.74 to 5.43 ± 0.55 mM within the obese group [21]. Another evaluation on the same subjects by Samjoo and colleagues found reductions in gynoid fat (36.47 ± 1.26 to 36.37 ± 1.38%), leg fat (30.98 ± 1.48 to 30.46 ± 1.62%) and leptin (12,966 ± 1787 to 8575 ± 1145 pg/mL) [21].

While it seems that exercise without weight loss may foster improvements through glycogen repletion and the loss of body fat, this has not always proven to be consistent. For instance, exercise training @ 60–70% of VO_2peak_ reduced plasma FFA (500 ± 42 to 429 ± 39 µmol/L), visceral adipose tissue area (164.3 ± 18.3 to 143.6 ± 18.7 cm^2^, and hepatic triglyceride content (8.55 ± 2.49% to 6.79 ± 1.90%) but did not promote reductions in HOMA-IR scores following the intervention period. Notably, the aerobic exercise intervention consisted of 30 to 45 minute sessions only 3 times per week, which had no influence on HOMA-IR scores (4.59 ± 0.69 to 4.4 ± 0.76) [23]. It should be recognized that the HOMA-IR assessment does not always track slight but significant changes in insulin sensitivity [16].

Interestingly, when moderate (50% of VO_2peak_) and vigorous (70% of VO_2peak_) intensity exercise were directly compared by eliciting a similar expenditure of energy (1000 kcals/week) for 12 weeks without weight loss, the vigorous intensity group experienced a significant reduction in VAT (−39 ± 11 cm^2^) compared to no significant change with moderate intensity exercise. The beneficial influence of vigorous intensity exercise on VAT corresponded with previously published improvements in insulin sensitivity with this type of training [25]. Therefore, while vigorous intensity exercise may influence glycogen utilization and repletion, the impact of vigorous intensity exercise on VAT may also contribute to improvements in insulin sensitivity as well.

### Influence of interval exercise training

Since intensity and duration play an important role in energy metabolism, it would seem logical to assess the impact of highly vigorous exercise training or interval exercise training on insulin resistance. In a 7 week highly vigorous (~80% of HR_max_) aerobic exercise intervention that progressively increased sessions per week (ie., from 3×/week to 5×/week) and duration per session (from 30 min to 60 min), there was a collective 15.2 kg loss of fat mass. Although no significant weight loss occurred, the reduction in fat mass translated into a 7.3% increase in ISI-M for the control group and a 29.2% increase in ISI-M for the prediabetic group [28].

In studies utilizing interval training (i.e., alternating bouts of a 30 second all-out sprint) on a cycle for only 2 weeks, the short term intervention promoted reductions in waist circumference (−1.1%) and hip circumference (−1.0%) and was associated with a 23.2% increase in ISI 24 hours following the last exercise session. Additionally, resting fat oxidation was higher (+18.2%) at 24 hours, but returned near baseline levels at 72 hours following the last exercise session. Unfortunately, ISI also returned to near baseline levels at 72 hours suggesting a potential transient alteration in glycogen alteration being responsible for the exercise-induced improvement in ISI at 24 hrs post training [5]. Similarly, when aerobic exercise of vigorous intensity (65% of VO_2peak_) was compared to shorter durations of interval training (ie., 10 second cycle sprints against 0.05 kg per body weight resistance) during a 2 week period, there were no really differences in the influence of the intervention on insulin resistance (assessed by HOMA-IR) in the aerobic exercise group (3.27 ± 0.11 to 3.15 ± 0.45) and the interval training group (2.88 ± 0.24 to 3.03 ± 0.22), respectively. Since the 30 minute sessions held 5 days a week of aerobic exercise and 8 to 12 repetitions of interval training only lasted two weeks, this may have been an insufficient stimulus to reduce insulin resistance, but was able to detect the relatively acute influence of exercise training on glycogen repletion [22].

## Exercise-Induced Weight Loss

Since exercise without weight loss is capable of enhancing insulin-stimulated glucose disposal especially when accompanied by a reduction in VAT, it seems intuitively obvious that insulin sensitivity would improve with exercise-induced weight loss. In many cases, weight reduction via exercise is accompanied by reductions in visceral adipose tissue, which seem to drive improvements in insulin resistance. For example, a 12 week aerobic exercise intervention of progressive vigorous intensity (60–65% to 80–85% of HR_max_) most days of the week (5 sessions/week) for 50 to 60 minutes a session induced approximately 8% weight reduction with significant reductions in VAT amongst all groups with various types of glucose tolerance. In particular, there was a significant reduction in group lost a significant amount of VAT in individuals with Impaired Glucose Tolerance (IGT) (ie., −75 cm^2^), with Normoglycemic (NGT) Group (ie., −46.9 cm^2^),individuals with Impaired Fasting Glucose (IFG) (ie., −53.3 cm^2^), individuals with Combined Glucose Intolerance (CGI) (−15.5 cm^2^). These reductions were all accompanied by improvements in glucose tolerance demonstrated by reduced plasma glucose at 120 minutes of an OGTT in all groups. Additionally, insulin sensitivity and β-cell function, assessed by a hyperinsulinemic-euglycemic clamp procedure, was enhanced following the intervention within all groups highlighting the multifaceted benefits of vigorous exercise that promotes significant weight loss [29].

Interestingly, slight reductions, but still significant, in body weight or body mass index may mitigate insulin resistance. For example, an aerobic intervention lasting 16 weeks of vigorous intensity (75% of VO_2peak_), performed three to five sessions per week for 45 minutes each session promoted a 10% reduction in fat mass that correlated with an improvement in the glucose infusion rate (r=−0.635) used to assess insulin sensitivity. More specifically, insulin sensitivity was assessed 36–48 hours following the last exercise session via the hyperinsulinemic-euglycemic clamp procedure and revealed an ~23% increase in the glucose infusion rate during steady state period at the end of the procedure (Dube et al. 2012). A similar aerobic exercise intervention lasting 16 weeks of vigorous intensity (60–80% of APMHR) just two to three sessions per week for 60 minutes each session reported reductions in body mass index throughout the intervention from 24.6 ± 3.4 kg/m^2^ to 24.0 ± 3.4 kg/m^2^ at week 8 and 23.9±3.4 kg/m^2^ at week 16. While BMI is a nonspecific measure of adiposity and was only slightly reduced in these studies, the reduction was significant and accompanied by increases in adiponectin from 4.9 ± 2.4 to 7.5 ± 3.9 µg/mL at week 16. Additionally, these changes significantly improved lipid profiles and reduced insulin resistance, as assessed by HOMA-IR, from 2.2 ± 1.1 to 1.8 ± 1.1 at week 16 [30].

### Exercise intensity and weight loss: Impact on insulin sensitivity

It might be argued that exercise of a specific duration will improve glucose metabolism regardless of the prescribed intensity. While moderate intensity exercise may be capable of promoting weight loss and thus improving insulin resistance, studies have demonstrated that the impact of vigorous intensity exercise training may be more effective in mitigating insulin resistance. For instance, in two particular [31,32], the total volume of exercise was defined by miles traveled each week and the intensity established for each group determined how long each session lasted. Specifically, each study used a method of low volume/moderate intensity (~12 miles/week, 1,200 kcal/week, 50–55% VO_2peak_), low volume/vigorous intensity (~12 miles/week, 1,200 kcal/week, 65–80% VO_2peak_), and high volume/vigorous intensity (~20 miles/week, 2,000 kcal/week, 65–80% VO_2peak_). The duration was calculated weekly for each group, which was similar between the low volume/moderate intensity and high volume/vigorous intensity groups (~50–60 minutes). In the study by Slentz and colleagues, body mass was reduced within each group but VAT was only reduced in the high volume/vigorous intensity group [32]. Remarkably, insulin sensitivity index (assessed by a 3 hour intravenous glucose tolerance test and Bergman’s minimal model to calculate SI) improved within all groups but β-cell function (calculated as Disposition Index) was greater in the moderate intensity group compared to the vigorous intensity groups.

In the study by Bajpeyi and colleagues, they employed multiple variations of chronic exercise training lasting for 8 months and ranging from low volume/moderate intensity (1,200 kcal/wk at 40–55% peak O_2 consumption_), low volume/high intensity (1,200 kcal/wk at 65–80%V˙O_2peak_), and high volume/high intensity (2,000 kcal/wk at 65–80% V˙O_2peak_) [31]. Compared to the control group, all interventions promoted modest weight loss (1.1 kg). While insulin sensitivity (assessed by a 3 hour intravenous glucose tolerance test and Bergman’s minimal model to calculate SI) was increased in all groups at 16–24 hrs post last exercise bout, the improvements in insulin sensitivity only continued in the low volume/moderate intensity group (+35.2 ± 1.3%) and the high volume/vigorous intensity group (+27.4 ± 0.7%). Even though all of the interventions lasted for 8 months, the immediate post intervention improvement in insulin sensitivity in the low volume/high intensity group (+6.0 ± 0.9%) was not retained. Mitochondrial density and citrate synthase activity was only significantly higher within the high volume/vigorous intensity group, which might have fostered even more efficacious alterations in glucose metabolism [31]. On the other hand, it is quite surprising that the change in body weight in the high volume/high intensity group were similar to those exhibited by the low volume/moderate intensity group. Assuming a caloric deficit of 2,000 kcal/week in the high volume/high intensity group, this should have promoted a total amount of weight loss that was fifteen fold higher than that reported in this study.

When moderate intensity (50–60% of HRR) aerobic exercise was examined during a 6-month intervention that consisted of 45 minute sessions on three occasions per week, a significant reduction in weight was observed in individuals without metabolic syndrome (−7 ± 4%) and individuals with metabolic syndrome (−5 ± 5%) subjects. Additionally, there were reductions in fat mass (−11 ± 8%) and VAT (−9 ± 19%) in the metabolic syndrome group similar to the non-metabolic syndrome group. Although HOMA-IR scores were not significantly reduced, plasma glucose at 120 minutes of an OGTT was lowered (−11 ± 16%) in individuals with metabolic syndrome. Reductions in inflammatory markers such as high-sensitive C-reactive protein (−25 ± 40%) and tumor necrosis factor alpha (−14 ± 25%) also occurred following the intervention in the metabolic syndrome group [33].

While aerobic exercise is the common choice of modality for intervention programs with glucose intolerant individuals, current recommendations for physical activity include both aerobic and resistance exercises. In two separate studies by Donges et al. 2013 and Slentz et al. 2011, they implemented similar exercise interventions of aerobic exercise, resistance training, and a combination of the two [34,35]. In the study by Donges et al., they included aerobic exercise of vigorous intensity (75–80% APMHR) with progressing duration (40–60 minutes/session), resistance training of 9 exercises progressively performed (3 sets/10 repetitions to 4 sets/8 repetitions at approximately 75–80% of 1RM), and a combination of half the aerobic exercise and resistance training three times each week [34]. Although ISI-M was enhanced within each group following the intervention period, only the aerobic group experienced significant weight loss (−1.9 ± 0.7%) accompanied by reductions in fat mass (−4.5 ± 1.6%). However, each group experienced similar significant reductions in visceral adipose tissue (~10% each) and subcutaneous adipose tissue (~4% each) [34]. In the study by Slentz and colleagues, body weight significantly reduced within the aerobic group (−2.0 ± 3.8%) and the combination group (−2.1 ± 3.2%) with visceral adipose tissue reductions only occurring in the aerobic group (−15.9 ± 34 cm^2^), which is somewhat surprising considering equivalent improvements in the HOMA-IR scores within each aerobic (−0.40 ± 0.8) and combination (−0.50 ± 0.9) groups [35]. Additionally, the improvements in insulin sensitivity following the study by Donges and colleagues were accompanied by reductions in inflammatory markers of tumor necrosis factor alpha and interlukin-6 among each group [34]. The impact of weight loss on liver fat of −2.5 ± 5.7 Hounsfield units demonstrates an additional benefit of exercise-induced weight loss on the mechanisms responsible for insulin resistance [35].

## Caloric Restriction and Exercise with Weight Loss

It seems evident from our previous studies that the volume of exercise required to elicit a reduction in adipose tissue or induce weight loss with improvements in insulin sensitivity is quite cumbersome. For example, in our studies we recognized that for a middle-aged 82 kg female with a VO^2peak^, exercise-induced weight loss of 0.5 kg/week necessitated 90 minutes cycle ergometer 5 days a week. As a result of this demanding volume of exercise training, dietary modification or caloric restriction are commonly implemented and utilized to reduce insulin resistance.

While caloric restriction-induced weight loss may have a large influence on insulin sensitivity, the subsequent loss of fat free mass may compromise glucose uptake, given that skeletal muscle is responsible for a larger portion of glucose uptake. This is observed in a study that implemented caloric restriction alone (−400 kcal/day or 2800 kcal/week) as well as with moderate (45–50% of HRR) and vigorous (70–75% of HRR) intensity exercise of progressing durations (moderate intensity=20–25 to 55 minutes/session, vigorous intensity=10–15 to 30 minutes/session) three days a week for 20 weeks. Although average weight loss was 13.4 ± 4.6% and included an approximate 25% reduction in visceral adipose tissue among all groups, plasma glucose at 120 minutes of an OGTT was unchanged. More importantly, fat and lean mass was reduced in each group but relative lean mass was reduced to a greater extent in the caloric restriction group (−36.2 ± 17.1%) compared to the moderate intensity exercise with caloric restriction group (−27.7 ± 14.8%) and the vigorous intensity exercise with caloric restriction group (−26.7 ± 12.1%). However, relative aerobic capacity increased within each group but significantly greater within the vigorous intensity exercise with caloric restriction (+24.2 ± 27.6%) group [36].

When fat free mass was preserved following a 12 week aerobic exercise intervention with or without a caloric deficit (500 kcals/day), insulin-stimulated glucose disposal was significantly improved (20–28% increase in relative). The exercise included progressing vigorous intensity (60–65 to 80–85% HR_max_) for 50–60 minutes most days of the week (5 days/week) but the exercise alone group was eucaloric for the duration of the study. Weight loss occurred in both the aerobic exercise (3.8%) and aerobic exercise with caloric restriction (7.4%) group that was accompanied by similar reductions in abdominal adiposity and improvements in insulin sensitivity (~30%) [37].

Since weight loss typically enhances insulin sensitivity especially when skeletal muscle is retained, it seems logical that resistance training with weight loss will improve in the same manner. In an intervention consisting of 6 exercises with progressing intensity (2–3 sets/15 repetitions progressed to 3–4 sets/10–12 repetitions) three days a week for 6 months, resistance training was combined with caloric restriction (−624 ± 133 kcals/day) and was compared to caloric restriction alone (−621 ± 128 kcals/day). Although significant reductions occurred in body mass visceral adipose tissue, abdominal subcutaneous adipose tissue occurred, no significant changes were observed for relative glucose disposal. Additionally, the intervention actually resulted in significant reductions in mean total lean body mass within the caloric restriction and resistance training with caloric restriction groups [37].

Interestingly, another resistance training intervention lasting 12 weeks implemented 4 exercises at a low intensity just one day a week for 40 minutes while a 1600 kcals/day diet was provided. Notably, there was no significant weight loss but reductions in total cholesterol (−14.5 g/L) and triglycerides (−11 g/L) occurred. Although reductions of inflammatory markers were observed, insulin resistance remained, as HOMA-IR scores were not affected [38]. Unfortunately, it seems resistance training, much like aerobic exercise, requires sufficient stimulus to drive reductions in body weight or fat mass and affect insulin resistance. However, the physiological responses from resistance training versus aerobic exercise or both together on insulin resistance remain unclear.

A comparative study of aerobic exercise, resistance training, and caloric restriction has also been investigated. Aerobic exercise included a range of vigorous intensity (67–80% of HR_max_) with progressing duration (20 to 40 minutes/session) three days per week. Whereas the resistance training included 10 exercises performed at 80% of 1RM (1–2 sets/10 repetitions) three days per week. The exercise groups also took part in the 800 kcals/day meals that were also provided to the caloric restriction group. The goal of the exercise and/or caloric restriction was to reach a body mass index less than 25 kg/m^2^. Following weight loss, subjects were instructed to maintain a balanced diet and the exercise groups continued activity until 1 year follow-up measurements. Although significant weight loss occurred, weight regain was observed within each group. All groups experienced significant reductions in intra-abdominal and subcutaneous adipose tissue following weight loss with increases after a year. Although lean mass was reduced in both aerobic and caloric restriction groups, insulin sensitivity was significantly improved within all groups after weight loss, but only continued to improve within the aerobic exercise group after weight loss and the one year evaluation [38].

Interestingly, it seems that a 10% reduction in body weight following an intervention has a dramatic influence on insulin sensitivity. For instance, in a study that elicited a 10% reduction in body weight from caloric restriction (500–1000 kcals/day) or combined exercise with caloric restriction, significant increases in ISI-M were observed. The progressive aerobic exercise intensity (70% to 85% of HR_peak_) for 30 minutes and resistance training (65% to 80% of 1RM, 1–2 sets/8–12 repetitions to 2–3 sets/6–8 repetitions) three days per week with caloric deficit reduced body weight (−10±2%) and intrahepatic fat (−45 ± 8%) similar to the caloric restriction group. These reductions were accompanied by a 66 ± 25% and 68 ± 28% increase in ISI-M for the caloric restriction group and combined group, respectively [39].

Similarly, a study by Schenk and colleagues compared the effects of caloric restriction alone (500–800 kcals/day) or with vigorous intensity (85% of HRmax) aerobic exercise (3–4 sessions per week for 45 minutes each) after a 12% reduction in weight was elicited [40]. The aerobic exercise with caloric restriction group reached 12% weight loss quicker (20 ± 2 weeks) than the caloric restriction group (30 ± 3 weeks), and was followed by a weight maintenance period. Although the aerobic exercise with caloric restriction group demonstrated increased levels of resting whole-body fatty acid oxidation more than 20%, insulin sensitivity increased similarly by 60–70% within both groups [40]. Additionally, skeletal muscle pro-inflammatory c-Jun N-Terminal Kinase (JNK) and fatty acid mobilization and uptake were 40% and 30% lower, respectively [40]. These reductions were likely due to a significant loss of fat mass altogether.

The importance of significant weight loss was also supported by a study from Mason and colleagues that compared the amount of weight loss relative to changes in HOMA-IR scores [41]. The intervention included aerobic exercise alone (70–85% of THR 5 days/week for 45 minutes each) or with caloric restriction, compared to caloric restriction alone to induce 10% weight reduction followed by 6 months of maintenance. Although weight loss was significant within the aerobic exercise group (−2.4%), the aerobic exercise with caloric restriction and caloric restriction groups demonstrated further weight loss (−10.8% and −8.5%, respectively) and significant HOMA-IR score reductions (−26% and −24%, respectively). The percent weight loss correlated with HOMA-IR score reductions with an r value of −0.23 for <5%, −0.69 for >5–10%, and −1.10 for >10% weight loss [41].

However, the intervention responsible for the weight loss may elicit different physiological responses. For instance, weight lost solely from dietary intake reduction may affect fat depots but weight lost from a combination of both dietary intake reduction and increased energy expenditure may improve muscle metabolism while reducing adiposity. This concept is demonstrated in a study by Toledo and colleagues that compared caloric restriction (reduce intake by 25%) to that with aerobic exercise (60–70% of HR_max_ for 30–40 minutes 3–5 days/week) to induce at least a 7% reduction in weight over a 16 week period [42]. Fortunately, each group experienced ~10% weight loss accompanied by ~19% reduction in fat mass, but a ~17% of visceral adipose tissue in the aerobic exercise with caloric restriction group as opposed to the ~25% reduction in the caloric restriction group. However, the aerobic exercise with caloric restriction group demonstrated a 49 ± 16% increase in mitochondrial density supporting an improved mitochondrial oxidative capacity, versus the 17 ± 4% reduction in mitochondrial size observed in the caloric restriction group [42].

Comparatively, in studies by Ryan et al. 2012 and Strasnicky et al. 2009, both interventions of similar structure comparing vigorous intensity aerobic exercise with caloric restriction to caloric restriction itself induced ~8% weight loss [43,44]. However, Strasnicky and colleagues grouped the responses together, given that the weight loss was similar between the groups. Both groups experienced significant losses in fat mass (−6.4 ± 0.6 kg) and waist circumferences (−8.6 ± 0.8 cm) that was accompanied by a significant improvement in ISI-M of ~45% [44]. Additionally sympathetic responsiveness was assessed by measuring norepinephrine spill over rate during an OGTT using a radioisotope dilution method. Weight loss promoted resting norepinephrine to decrease, but only the aerobic exercise with caloric restriction group experienced significant sympathetic responsiveness to glucose at 90 minutes of OGTT. This suggests weight loss may also reverse the blunted sympathetic responsiveness to glucose ingestion commonly seen in glucose intolerant individuals [43]. Whereas in the study by Ryan and colleagues, significant reductions in fat mass (−14%), visceral fat area (−13%), and subcutaneous abdominal fat area (−12%) were observed within each group [44]. This supported the overall improvement in glucose utilization and non oxidative glucose disposal during the hyperinsulinemic-euglycemic clamp procedure, 14% and 24% respectively. However, insulin-stimulated glycogen synthase activity was significantly higher in the aerobic exercise with caloric restriction group of impaired glucose tolerant individuals only. This suggests that for individuals with glucose intolerance, weight loss from caloric restriction combined with aerobic exercise improves fitness that may have an effect on insulin to increase glycogen synthase activity greater than weight loss from caloric restriction alone [44].

Under rare circumstances, weight loss from a combined effort of dietary modification with exercise may not result in an enhanced insulin sensitivity. For instance, a study by Oh and colleagues examined various forms of exercise (Tae-Bo, Yoga, Walking) for 6 months that consisted of various intensities performed 2 to 3 days per week for 40 minutes while maintaining a diet less than 1500 kcals/day [45]. Although significant weight loss occurred at 6 months (−5 kg) and at a one year re-evaluation (−4.3 kg), insulin resistance, as assessed by HOMA-IR, remained unchanged [45]. Although this intervention was new to a group of community residing individuals, the prescribed amount of exercise seemed inadequate and uncontrolled.

### Differences in weight loss

A collective amount of studies using the same fixed amount of vigorous intensity aerobic exercise with or without caloric restriction have been able to identify differences in responses from the context to which the weight was lost. For example, several studies have utilized 12 weeks of vigorous intensity (65–75% of VO_2max_) aerobic exercise five days a week for 50 to 60 minutes [46–49]. In this study by Haus and colleagues, they observed significant reductions in weight within each group, but 4.7 kg more in the aerobic exercise with caloric restriction group. Total abdominal fat was reduced in the aerobic exercise group (−9 ± 4%) but more in the aerobic exercise with caloric restriction (−20 ± 4%) [46]. Total subcutaneous fat, superficial, deep subcutaneous and visceral adipose tissue was reduced similarly between groups. Suppression of hepatic glucose production was examined during a two-stage hyperinsulinemic-euglycemic clamp that included an infusion of artificial lipids to simulate baseline levels of circulating free fatty acids. Each group demonstrated a significant improvement to suppress hepatic glucose production during hyperinsulinemia (−45 ± 22% in aerobic exercise and −50 ± 20% in aerobic exercise with caloric restriction) but the aerobic exercise with caloric restriction group had greater suppression of hepatic glucose production than the aerobic exercise group when artificial lipids were infused [46].

Additionally, the study by Kelly and colleagues revealed more weight loss and fat mass loss in the aerobic exercise with caloric restriction group, which is practical. However, ISI-M was only significantly increased (2.69 ± 0.5 to 4.16 ± 0.55) in the aerobic exercise with caloric restriction group [49]. There was also a significant reduction in glucose-dependent insulinotropic polypeptide in response to an OGTT in the aerobic exercise and caloric restriction group. This finding was attributed to improving gut peptide release, which may help mediate glucose-stimulated insulin responses [49].

However, in one study by Solomon and colleagues, significant reductions in leptin were observed for the aerobic exercise group (−12.2 ± 3.8%) but greater in the aerobic exercise with caloric restriction group (−31.6 ± 6.0%) were accompanied by similar reductions in weight and fat mass. Interestingly, insulin-stimulated glucose disposal, as assessed by the hyperinsulinemic-euglycemic clamp procedure, was significantly greater in both the aerobic exercise group (55.1 ± 19%) and the aerobic exercise with caloric restriction group (65.1 ± 14.4%) of similar nature [47]. Although greater body compositional changes occurred in the aerobic exercise with caloric restriction group, the changes in insulin sensitivity seem to be driven by the exercise. Similarly, the other study by Solomon and colleagues revealed greater body compositional changes in the aerobic exercise and caloric restriction group than the aerobic exercise group. However, the improvements in insulin-stimulated glucose disposal, assessed by the hyperinsulinemic-euglycemic clamp procedure, were similar within each group (30.7 ± 12.2% in aerobic exercise group and 31.5 ± 23.7% in the aerobic exercise and caloric restriction group). Interestingly, fatty acid oxidation was assessed as well and revealed the non plasma lipid derived contribution of free fatty acids in the basal state increased [48]. This suggests the utilization of lipids from fat depots and reduced adiposity, which was similar between both groups.

Commonly, aerobic exercise is coupled with caloric restriction and compared to exercise alone, in a eucaloric state. Comparing interventions in this context cannot definitively distinguish beneficial differences. However, in a study by Murphy and colleagues caloric restriction was compared to aerobic exercises which were controlled to elicit a similar deficit [50]. The aerobic exercise was to expend 16% of energy for 3 months, then 20% until the final 12 months. This consisted of vigorous intensity (~71% of HR_max_) most days of the week (6 sessions/week) for ~62 minutes each. The caloric restriction was to reduce intake by 16% for 3 months, then 20% (−318 kcals/day) for the duration of the intervention. Significant reductions in body weight and fat mass occurred similarly between groups, which was accompanied by similar improvements in ISI-M [50]. However, the aerobic exercise group experienced significant reductions in both intermuscular adipose tissue and visceral adipose tissue versus the caloric restriction group that experienced visceral adipose tissue reductions only. Interestingly, the reductions in visceral adipose tissue within the caloric restriction group correlated with changes in ISI-M (r=−0.64). Whereas only the reductions intermuscular adipose tissue within the aerobic exercise group correlated with reductions in ISI-M (r=−0.71) [50]. Since visceral adipose tissue and intermuscular adipose tissue is commonly linked to oxidative stress, exercise-induced weight loss may have advantage over weight lost from caloric restriction.

For example, in a well-controlled exercise and feeding study, aerobic exercise of moderate intensity (50% of VO_2peak_) was progressively increased (1000 kcals/week gradually to 2500 kcals/week) to elicit a similar deficit as caloric restriction (1000 kcals/week minus 500 kcals until 2500 kcals/week). A control group was included, as well as an aerobic exercise group that received calories to maintain body weight. Following a 4 week weight stabilization period, weight reduction was only significant within the exercise-induced weight loss and caloric restriction (~6% each) groups. Visceral adipose tissue was reduced in the exercise group (−17 ± 7 cm^2^), caloric restriction group (−36 ± 10 cm^2^), and two-fold greater in the exercise-induced weight loss group (−71 ± 15 cm^2^). Interestingly, the improvements in insulin-stimulated glucose disposal were similar between the caloric restriction (+2.4 ± 0.9 mg/kgFFM/min) and exercise-induced weight loss (+2.5 ± 0.4 mg/kgFFM/min) groups. Notably, the caloric restriction group experienced significant reductions in lean thigh tissue (−7±1 cm^2^) as opposed to the increase found within the exercise-induced weight loss (+7 ± 1 cm^2^) group. Furthermore, significant insulin-stimulated suppression of glucose production was observed in the exercise group (+12 ± 2%), caloric restriction group (+10 ± 2%) and 3 times greater in the exercise-induced weight loss group (+27 ± 2%) [51,52]. The findings from this study illustrates that similar weight loss from caloric restriction or exercise does not yield the same physiological responses. Exercise-induced weight loss in this context reduced adiposity and systemic insulin resistance most effectively.

## Summary

Significant improvements in insulin sensitivity seem largely driven by weight reduction. Achieving weight loss through caloric restriction or dietary modification may be less cumbersome than energy expenditure, but the adaptations are not necessarily equivalent. When adaptations following weight loss are compared between caloric restriction and exercise, improvements in insulin stimulated glucose disposal occur similarly with greater adaptations from exercise-induced weight loss. Additionally, exercise-induced weight loss stimulates mitochondrial oxidative capacity and impacts endogenous glucose production by significantly suppressing unnecessary gluconeogenesis. The efficacy of sustained improvements in glucose metabolism may be influenced by exercise intensity as it relates to changes in body composition. Dietary weight loss effectively lowers adipose tissue considerably, but does not influence muscle metabolism similar to exercise training. However, both interventions may be used together as a powerful weight reduction technique as well as a weight management tool and a mechanism proven effective to improve glucose metabolism.

## Key Factor

The ability to detect changes in insulin sensitivity is only as good as the assessment tool used. Specificity is a vital training principle necessary for exercise training and this concept is similar to detecting differences in insulin responsiveness. The hyperinsulinemic-euglycemic clamp is the gold standard for assessing insulin action, but its difficulty and cost commonly influences the use of other assessments to examine insulin action at baseline and following an intervention. Other assessments using fasting or an Oral Glucose Tolerance Test (OGTT) are typically inexpensive assessments and are commonly used in a clinic setting or epidemiological studies that identify glucose intolerance. Unfortunately, the OGTT was not designed to assess intervention-based changes in glucose metabolism The Insulin Sensitivity Index (ISI) calculations correlate quite well with the clamp but still may not be sufficient assessors of insulin action compared to the clamp [17]. Additionally, the Homeostasis Model Assessment (HOMA) equation fails to correlate with the clamp assessment, but is commonly used to assess insulin action in many investigations.

Although it may not be realistic for each investigator to use the clamp as their assessment tool, the assessment tool used during an intervention must be considered when evaluating the impact on clinical care. Therefore, it is important to consider interventions that did not induce changes in insulin action due to the limitations of the tool utilized to assess the endpoint.
